# Amoxicillin-Induced Drug Reaction With Eosinophilia and Systemic Symptoms (DRESS) Syndrome With Acute Onset of Diffuse Rash and Acute Kidney Injury (AKI)

**DOI:** 10.7759/cureus.51707

**Published:** 2024-01-05

**Authors:** Marianne Cortes, Kyoung W Cho, Nayeem M Chowdhury, Jeffri-Noelle Mays, Chan H Shin

**Affiliations:** 1 Medical School, Nova Southeastern University Dr. Kiran C. Patel College of Osteopathic Medicine, Fort Lauderdale, USA; 2 Endocrinology, University of California Irvine, Irvine, USA

**Keywords:** urticaria, nikolsky sign, drug rash, t-lymphocytes, regiscar, amoxicillin, drug reaction with eosinophilia and systemic symptoms (dress) syndrome

## Abstract

Drug reaction with eosinophilia and systemic symptoms (DRESS) syndrome is an uncommon and potentially fatal adverse drug reaction that can affect individuals with immunosuppression, viral reactivation, pharmacogenetic susceptibility, and recent exposures to new medications. Due to the ambiguous symptomology of DRESS syndrome along with a lack of diagnosis and treatment criteria, there can be delays in diagnosis and management. Here, we present a case of a 60-year-old female with an uncommon presentation of DRESS syndrome due to a less commonly implicated drug. We aim to bring awareness to the various presentations associated with DRESS syndrome and inform readers about current diagnostic and treatment modalities used today. In addition, this case serves to provide insights that further evidence is needed to have standardized guidelines in place to effectively diagnose and manage affected patients.

## Introduction

Drug reaction with eosinophilia and systemic symptoms (DRESS) is a severe adverse drug reaction associated with a skin rash, visceral organ involvement, lymphadenopathy, eosinophilia, and atypical lymphocytosis, occurring in about 0.9 to two patients per 100,000 per year [1]. Individuals at risk include those with immunosuppression, human herpesvirus-6 (HHV-6), and pharmacogenetic susceptibility [2,3]. DRESS typically manifests as a delayed hypersensitivity reaction with a two- to eight-week latency phase, with high-risk drugs, including aromatic antiepileptic agents and sulfonamides [1,2]. While little is known about the exact pathogenesis of DRESS, the mechanism of activated T-lymphocytes releasing interleukin-5 (IL-5) leading to the characteristic eosinophilia has been described [3]. The mortality rate for DRESS is as high as 10%, with most deaths attributed to severe organ involvement, most commonly of the liver and kidneys [1-3]. While there are current standardized guidelines for diagnosing and treating DRESS, they are not well validated. Through our case report, we aim to increase awareness on the presentation of DRESS and emphasize the need for timely diagnosis and treatment. In addition, our case is unique in that DRESS was induced by a lower-risk drug, amoxicillin, with a one-day latency period after initiation.

## Case presentation

**Figure 1:**
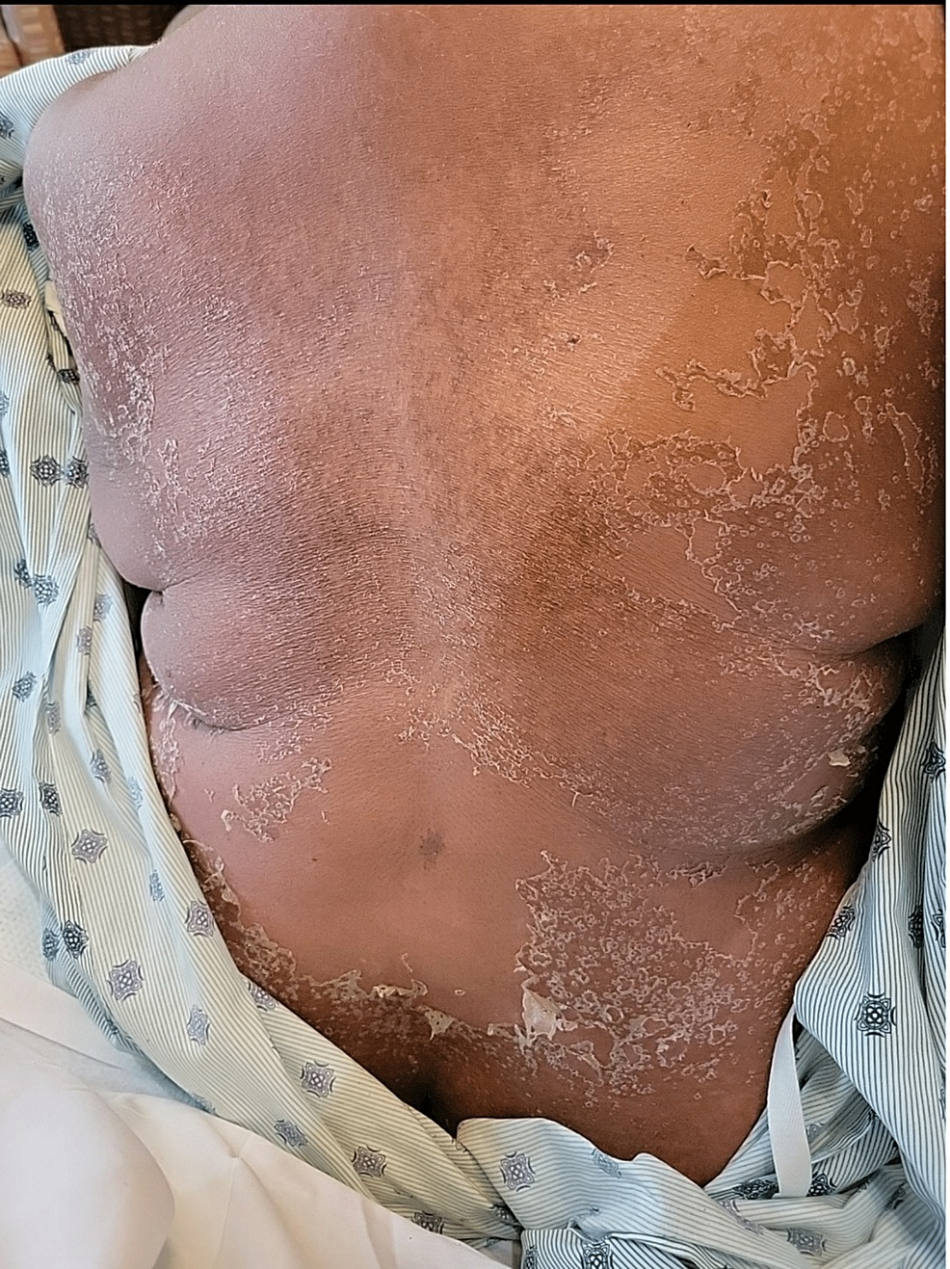
Dark red diffuse patch/plaque erythroderma sparing the skin folds. There is increased superficial epidermal desquamation evident. Dark brown to black macules diffusely present across back within areas affected by patches.

**Figure 2:**
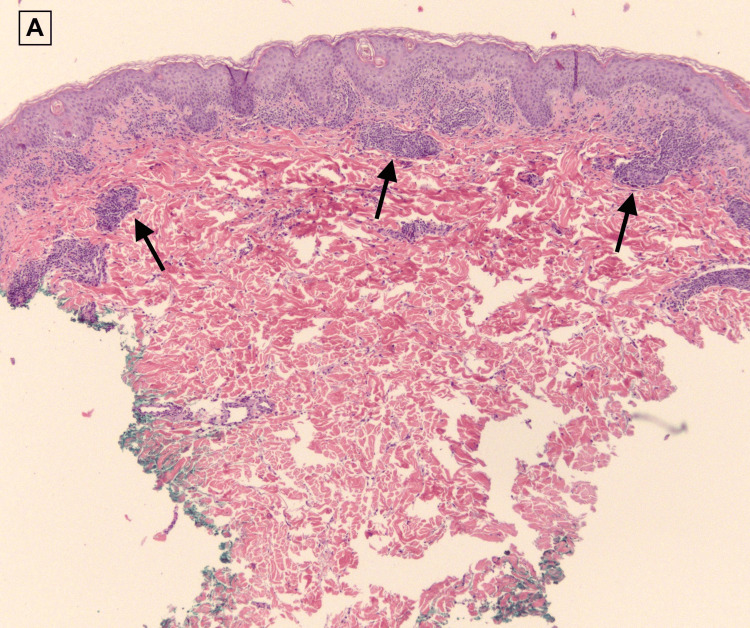
Superficial perivascular inflammatory infiltrate mainly composed of lymphocytes with eosinophils. Hematoxylin and eosin (H&E) 5x.

**Figure 3:**
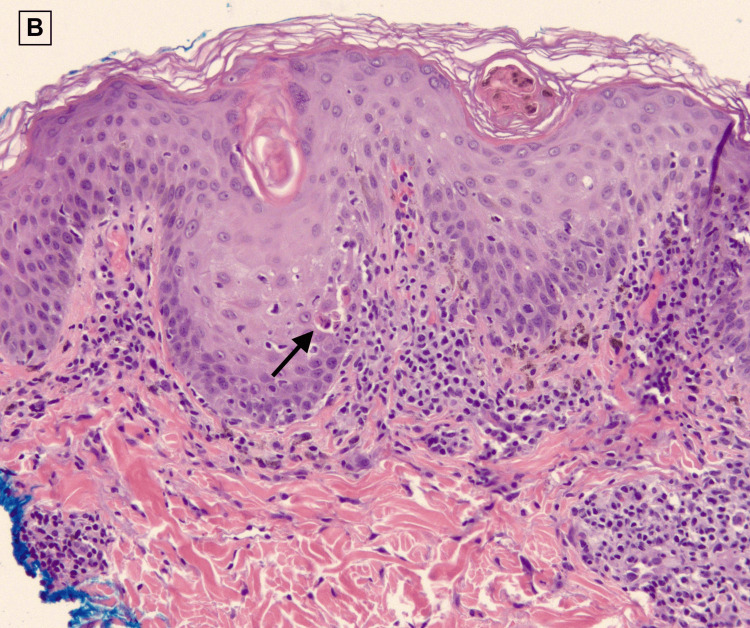
Epidermal dyskeratosis with diffuse vacuolar interface dermatitis in DRESS. Hematoxylin and eosin (H&E) 20x.

A 60-year-old female with a past medical history of hypertension, type 2 diabetes mellitus, and hyperlipidemia presented with two weeks of red, itchy, diffuse body rash in addition to a mild fever of 100.1°F. Two weeks prior to the admission, she was prescribed amoxicillin by her primary care physician for pharyngitis. The patient stated that immediately after taking the first dose of amoxicillin, she broke out a diffuse rash over her chest and proximal arms associated with itching and burning (Figure 1). Associated symptoms included fatigue, lymphadenopathy, non-bloody diarrhea, dark-colored urine, diffuse myalgia, shortness of breath, and dysphagia. A small mucosal lesion was evident on the left buccal area, and Nikolsky sign was negative. Her only medications were ibuprofen 800 mg as needed and amoxicillin 100 mg. Her laboratory findings depicted significant leukocytosis, erythrocytosis, thrombocytosis, hypernatremia, hyperkalemia, AST/ALT levels of 138/102 units/L, and BUN/creatinine levels of 142/5.8 mg/dl. A viral panel indicated presence of Epstein-Barr virus (EBV). Chest X-ray was unremarkable. She was subsequently admitted to the intensive care unit for airway protection, acute kidney failure, and further management. Viral, infectious, malignant, and rheumatologic causes were ruled out via blood work and cultures. Skin punch biopsies of the upper extremities, right abdomen, and right thigh were done, and pathology report depicted predominantly lymphocytic dermal inflammatory infiltrates with scattered eosinophils consistent with a drug eruption (Figures 2, 3).

The patient was started on pulse doses of methylprednisolone 125 mg for three days along with triamcinolone 0.1% cream for areas affected by the rash. Supportive measures were also given through fluid replacement and continuous monitoring of electrolytes and renal function. By the third day of hospitalization, the patient had drastic improvement in her renal function and rash. The patient was discharged after a total of one week.

## Discussion

As illustrated in our patient, some of the hallmark features of DRESS syndrome include cutaneous and mucosal membrane involvement. The characteristic rash can vary in appearance and consist of an urticarial, maculopapular eruption, while in some instances, it consists of bullae, pustules, vesicles, purpura, target lesions, fascial edema, cheilitis, and erythroderma [2]. In addition, systemic symptoms, including fever, tender lymphadenopathy, laboratory abnormalities (leukocytosis with eosinophilia and liver enzyme elevation), HHV reactivation, and manifestations of visceral involvement are common among patients with DRESS syndrome [1,3,4]. Organ involvement commonly affects the liver and is reported in 90% of cases, although acute interstitial nephritis, interstitial pneumonia, and myocarditis can also occur less commonly [1]. The life-threatening nature of this condition stems from the severity of organ involvement, with its mortality estimated to be around 10% [4]. In our case, acute renal failure proved that the systemic nature of the condition was present. Most cases also involve leukocytosis with eosinophilia and/or mononucleosis, which was not seen in our case, making the diagnosis of DRESS syndrome more difficult [1,4].

DRESS syndrome is a challenging entity with no reliable standard for the diagnosis as it relies on the diagnosis of exclusion. Differentials for our patient included DRESS, Sezary syndrome, lymphoma, human immunodeficiency virus (HIV), mononucleosis, and scarlet fever due to the recent pharyngitis episode. The proposed diagnostic criteria for DRESS are based on clinical and laboratory ﬁndings, with history of previous exposures being one of the most important findings [1,5]. While multiple diagnostic criteria have been proposed with limited success, the Registry of Severe Cutaneous Adverse Reactions (RegiSCAR) scoring system has been widely used to aid in the exclusion of DRESS syndrome and must add up to a score of less than two to be excluded and greater than five to be considered definite [2]. Some of the criteria included in the RegiSCAR scoring system are fever greater than 101.3°F, eosinophilia, skin involvement, organ involvement, and enlarged lymph nodes in at least two different areas and atypical lymphocytes [1-3,6]. In our case, a RegiSCAR score of 5 deemed the diagnosis of DRESS syndrome to be probable, which lead to punch biopsies of the lesions to be taken to confirm. Although the histopathologic features of DRESS are heterogeneous and nonspecific, characteristic patterns associated with DRESS include interface dermatitis with basal vacuolization in 75% of cases, eczematous pattern in 40%-75% of cases, and vascular damage with perivascular lymphocytic infiltrate in 50% of cases [1]. A majority of these findings were consistent in our case, leading to the final diagnosis of DRESS syndrome in our patient. 

There are no current evidence-based guidelines for the treatment and management of DRESS syndrome, but the most important first step is the immediate identification and withdrawal of the culprit drug as this leads to a better prognosis [2]. Supportive care including fluid, electrolyte, and nutritional support along with gentle skin care is recommended [1]. The adjunct use of topical corticosteroids and oral antihistamines may help in alleviating symptoms [1,5]. The use of oral corticosteroids has unknown efficacy and evidence, but a starting dose of 0.5 to 1 mg/kg/day of prednisone is frequently used and then gradually tapered off [2,3,5]. Follow-up is important in these patients to confirm resolution of systemic symptoms and monitor for reactivation of latent viruses [3].

Viruses in the herpes family are commonly associated with DRESS syndrome, with the most notable being HHV-6 and EBV, with our patient testing positive for EBV. This phenomenon is not widely understood, but it is suggested that reactivation of the virus due to an immunocompromised state causes an expansion of virus-specific and nonspecific T cells that eventually cross-react with certain drugs, similar to the mechanism of graft-versus-host disease [2]. This association of viral reactivation and DRESS syndrome has been reported in 75% of cases, with HHV-6 being the most common [1,6]. Due to these hypotheses, testing for viruses in this family is strongly recommended as it can be seen as a marker for chronicity in addition to a prognostic marker for disease severity [1].

While aromatic anticonvulsant drugs and allopurinol have been documented to be the most common causes of DRESS syndrome, amoxicillin has been previously linked to such a condition [1,6]. It is not thoroughly understood how less commonly implicated drugs, such as amoxicillin, cause DRESS syndrome, but it is hypothesized that these medications may directly increase HHV-6 and cytomegalovirus (CMV) replication [1]. Latency periods may differ depending on the type of causative agent, ranging up to 300 days [6]. The latency phase between the administration of amoxicillin and the onset of symptoms was one day in our patient, suggesting a possible unique feature of amoxicillin-induced DRESS syndrome. There has yet to be a report on such an immediate reaction as in our case. In previous cases depicting amoxicillin as the culprit for DRESS syndrome, there has been a correlation to an increased risk in patients showing an intolerance to sulfasalazine [6]. Although multiple factors may have contributed to the short latency period in our patient, this case emphasizes the need for further studies to be done to better understand the multiple etiologies of DRESS syndrome.

## Conclusions

Our report depicts a case of DRESS syndrome caused by a less common agent with a rapid latency period of one day, which has yet to reported in the literature. Due to this unusual presentation, the diagnosis of DRESS syndrome was not immediately assumed. While much remains unknown about DRESS, we would like to emphasize the importance of recognizing common signs and symptoms of this syndrome even if accompanied by other abnormal presentations. The multiple nonspecific symptoms that may present with DRESS syndrome along with a lack of standardized diagnosis criteria, and treatment has led to delays in management. As DRESS syndrome is initially a clinical diagnosis, healthcare providers must take a thorough history to detect any recent exposures. Due to its high mortality rate, it is crucial that clinicians develop a high index of suspicion for DRESS syndrome in order to treat effectively, especially in patients who have been prescribed antibiotics recently.
